# Economic Recession and Attendances in the Pediatric Emergency Department

**DOI:** 10.1155/2019/4186486

**Published:** 2019-02-10

**Authors:** Despoina Gkentzi, Vasiliki Katsoula, Sotirios Fouzas, Manolis Mentis, Ageliki Karatza, Gabriel Dimitriou

**Affiliations:** ^1^Department of Pediatrics, University of Patras Medical School, Patras, 26504 Rio, Patras, Greece; ^2^Department of Social Services, University General Hospital of Patras, 26504 Rio, Patras, Greece

## Abstract

The economic recession has been shown to have a negative impact on health services worldwide. The purpose of this study was to examine whether the recent financial crisis in Greece that started in 2009 has affected the attendances in the pediatric emergency department of a University Hospital covering for a large geographical area in Greece. The study was based on a retrospective analysis of the cases presented to the paediatric emergency department and compared the attendances in 2008 (i.e., before the beginning of the economic crisis) with those in 2013 and 2017. Data on demographics and characteristics of emergency department visits, such as timing, reason, and outcome were recorded for each child. There were a total of 35.572 children seeking examination in those three years and data were collected for 5662 (17.36%) of them. Overall, the attendance rate has increased up to 20% without an increase to the hospital admission rates which remained stable throughout the study periods. Between 2008 and 2017, the percentage of febrile children attending the ED increased by 33.8% and of those with respiratory disorders by 63.1%. Our results indicate that the need for pediatric hospital services has changed following the economic crisis which could reflect gaps in the primary care setting and could well also result from financial constraints.

## 1. Introduction

The economic crisis appeared in 2007 in the United States of America and after that spread to many European countries, including Greece. Greece has been severely affected by the financial crisis. The latter appears to have a negative impact on the quality of life of the citizens, as the most common effects of the crisis are unemployment, general insecurity, and declining expenses in the public sector, including budget cuts for healthcare [1]. The economic crisis inevitably poses a serious threat to human health and living conditions [2]. Mental health problems, communicable diseases, and suicides are increasingly more common in countries with poor economy including Greece [3–5]. Studies indicate that unemployment and job insecurity may increase the risk for the development of depression, suicide attempts, and suicide [1, 5–8].

Children are a particularly vulnerable population group, and a strong relationship between socioeconomic living conditions and children's health has been identified [9]. It has also been demonstrated that the economic crisis is resulting in low birth rates, especially when it is linked to rising unemployment [10]. Moreover, the adverse circumstances in which children live as a result of parental poverty may lead to poor child health. In addition, parents perceive that their children are in worse health than they are objectively, because of anxieties resulting from financial concerns. Unemployment leads to a reduction in the quality of family life (budget cuts in primary goods, public debts, reduced access to healthcare services, etc.), and also affects the mental health of parents [11]. The poor socioeconomic status of parents can affect children's psychopathology and have an impact on their physical wellbeing with short and long-term consequences [12, 13]. In our country, children are one of the population groups affected by the crisis [7, 14]. Prolonged exposure to early childhood poverty can have a substantial and potentially irreversible impact on the physical, mental, and social health of the young population [15]. Prolonged exposure to early childhood poverty has also been associated with a higher risk of developing chronic diseases in older age, such as cardiovascular [16] and Alzheimer's disease [17]. Few studies have been published to date on the impact of the current crisis on children's health [18–21].

In Greece, very few studies have focused explicitly on the impact of the economic crisis on children's health and have mainly concluded an increase in behavioral and mental health issues [22–25]. Few studies, acknowledging that these effects cannot be solely and directly attributed to the economic crisis, have indicated an increase in stillbirths as fewer pregnant women have access to prenatal care services [26] as well as an increase in the birth of small for gestational age infants [27]. To the best of our knowledge, the impact of economic crisis on the hospital attendances has not been investigated to date. In the present study, we aim to investigate for the first time in Greece the impact of the economic crisis on the pediatric attendances to the pediatric emergency department (ED) of a large tertiary hospital in Greece.

## 2. Materials and Methods

This retrospective study conducted in the Pediatric Emergency Department of the University General Hospital of Patras, Greece; this is a teaching hospital that provides tertiary services for the geographical area of the Region of Western Greece (population coverage approximately 1,000,000 or one-tenth of the total Greek population) including urban and rural areas. The study periods were the years 2008, 2013 and 2017 and in particular the first ten days of the first six months (January to June) of each year. The year 2008 was selected as representative of the pre-crisis period. The years 2013 and 2017 were selected as representatives of the years of economic recession (2009-2017). Of note, all public hospitals in Greece offer services for free and there is no direct cost to the patient.For the study purposes, data were collected by a trained researcher who reviewed the medical records of the ED using a structured questionnaire. Patients' demographics (sex, age, nationality, and place of residence), time of ED visit, primary symptoms, laboratory investigations, and outcomes were recorded in a dedicated database and analyzed.

Categorical variables were compared using the chi-square test, including trend analysis to investigate any trend in changes among the three surveys. Continuous variables were compared using one-way ANOVA. The level of statistical significance was set at 0.05 while all analyses were performed using the IBM SPSS software version 24.0 (IBM Corp., Armonk, NY, USA). The study was approved by the Institutional Review Board (IRB) of the University Hospital of Patras.

## 3. Results

The total number of children who attended the pediatric ED in 2008, 2013, and 2017 was 32.572; 10,015 children visited the ED in 2008, 10,977 in 2013, and 11,580 in 2017. The sample studied in the present survey was 5,662 children, which represents 17.38% of the total number of attendees. In particular, 1738 children were surveyed in 2008 (representing 17.35% of total ED visits for the respective year), 1823 (16.6% of ED visits) in 2013, and 2101 (18.14% of ED visits) in 2017.

**Figure 1:**
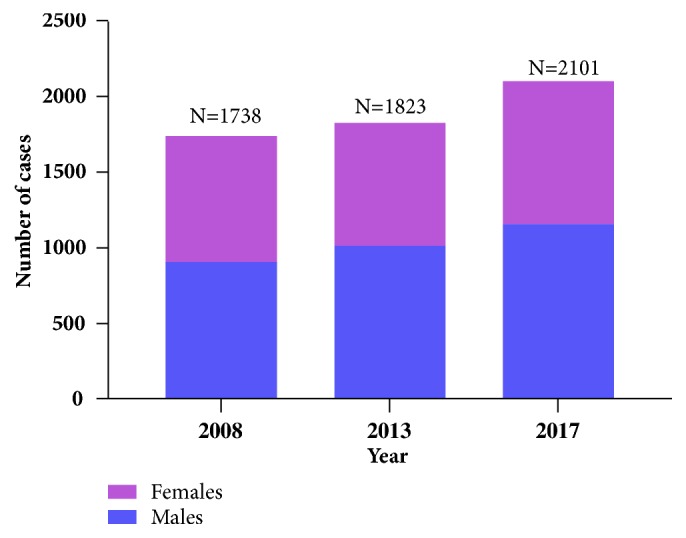
Number of cases and distribution by sex.

The number of children who visited the pediatric ED during the same period of the year increased by 4.9% between 2008 and 2013 and by 15.2% between 2013 and 2017. The increase in pediatric ED visits between 2008 and 2017 was 20.1% (Figure 1). Males were 905 (52.1%) of cases in 2008, 1011 (55.5%) in 2013, and 1155 (55%) in 2017 (Figure 1). There were no differences in sex distribution among the three surveys (chi-square P = 0.090; P-for-trend = 0.084).

**Figure 2:**
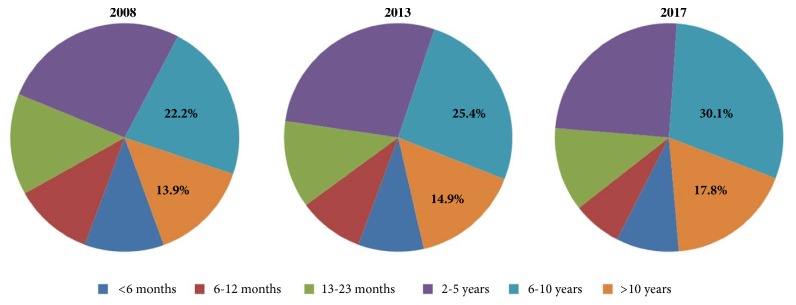
Distribution of ED attendees by age.

The age of children attending the pediatric ED increased from 5.2 ± 3.9 years in 2008 to 5.4 ± 3.9 years in 2013 and 6.1 ± 4 years in 2017 (one-way ANOVA P = 0,037). The distribution by age (Figure 2) also differed significantly among the three years (chi-square P < 0.001). In particular, the percentage of children 5-to-10 years old who visited the ED increased from 22.2% in 2008 to 25.4% in 2013 and 30.1% in 2017. Similarly, the percentage of children >10 years old increased from 13.9% to 14.9% and 17.8%, respectively. Overall, the percentage of children aged more than five years increased from 36.1% in 2008 to 40.3% in 2013 and 47.9% in 2017 (P-for-trend < 0.001) (Figure 2).

**Figure 3:**
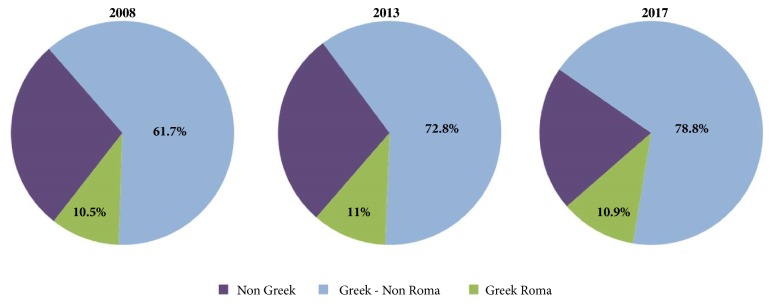
ED visits according to nationality.

The majority of children attending the ED were of Greek nationality: 72.2% (N = 1255) in 2008, 72.8% (N = 1327) in 2013, and 78.8% (N = 1655) in 2008 (Figure 3). The above trend was significant and increasing (chi-square *Ρ* < 0,001, P-for-trend < 0,001). The percentage of attendees belonging to Roma communities remained stable: 10.5% (N = 182) in 2008, 11% (N = 200) in 2013, and 10.9% (N = 230) in 2017 (chi-square P = 0.648) (Figure 3).

The place of residence of ED attendees is presented in Table 1. There was a constant increase in the number of children from the Local Regional Unit of Achaia (37.6% increase from 2008 to 2017; chi-square *Ρ* < 0,001, P-for-trend < 0,001). The number of cases referred to our hospital by other healthcare units of the Region of Western Greece remained stable, while the number of children of whom families were visiting the region of Western Greece at the time of ED presentation decreased significantly (60.2% decrease from 2008 to 2017; chi-square *Ρ* < 0,001, P-for-trend < 0,001) (Table 1).

**Figure 4:**
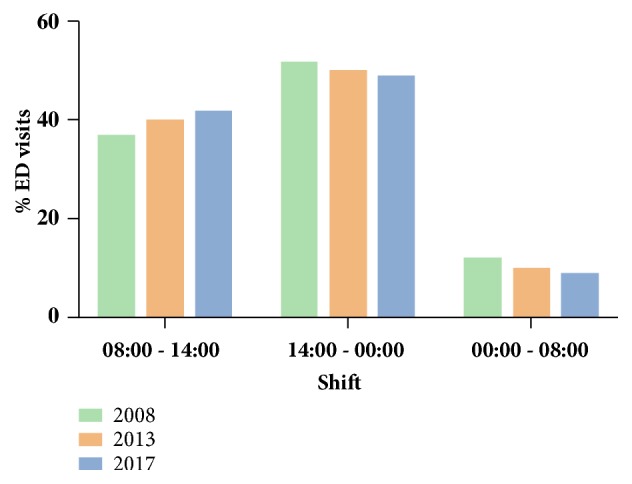
Time of ED visits.

There was a constant increase (as both crude number and percentage) of cases visiting the ED during the regular “morning” shift (Figure 4); between hours 08:00 and 14:00, 629 children attended the ED in 2008 (36.2% of ED visits), 725 in 2013 (39.7% of ED visits), and 847 in 2017 (40.3% of ED visits) (chi-square P = 0.021, P-for-trend = 0.020). From 2008 to 2017, the number of cases visiting the ED during the “morning” shift increased by 34.7%. The respective figures for cases visiting the ED during “afternoon” and “night” shifts decreased, albeit not significantly (P-for-trend = 0.128 for the “afternoon” shift and P-for-trend = 0.071 for the “night” shift) (Figure 4).

The number of patients transferred by ambulance was small and remained constant among the three surveys (2008: N = 15, 0.9% of ED visits; 2013: N = 16, 0.8% of ED visits; 2017: N = 27, 1.3% of ED visits; chi-square P = 0.326).

The main causes of presentation to the ED are shown in Table 2. There was a constant increase in the percentage of children with fever, from 26.5% in 2008 to 28% in 2013 and 29.4% in 2017 (chi-square P < 0.001, P-for-trend < 0.001). Similarly, the percentage of children with respiratory disorders increased from 14% to 16.5% and 18.9%, respectively (chi-square P < 0.001, P-for-trend < 0.001). Between 2008 and 2017, the percentage of febrile children attending the ED increased by 33.8% and of those with respiratory disorders by 63.1%. The number of children presenting to the ED due to other causes remained constant among the three surveys (Table 2). There was also a constant increase in the number of children visiting the ED to perform scheduled paraclinical investigations (i.e., blood sampling, X-rays, ultrasonography, etc.) and those attending the ED to obtain various health certificates. The percentage of such cases increased from 7.2% in 2008 to 8.5% in 2013 and 11.1% in 2017 (chi-square P < 0.001, P-for-trend < 0.001). The overall increase between 2008 and 2017 was 84.9% (Table 2).

**Figure 5:**
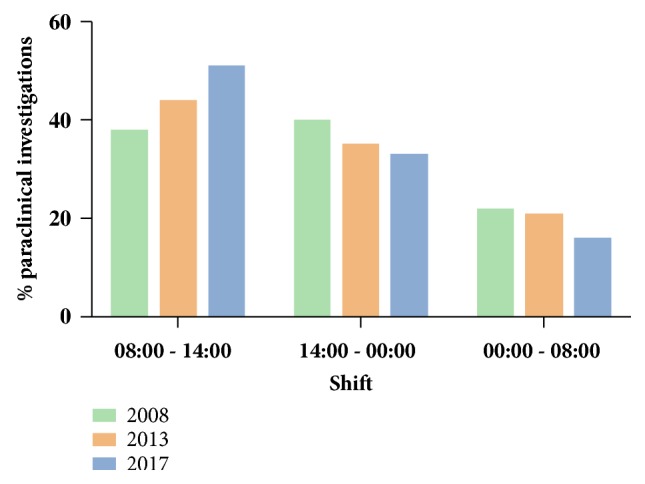
Paraclinical investigations according to the time of ED visit.

The percentage of children who required paraclinical investigations (laboratory and/or imaging) increased from 21.9% (N = 380) in 2008 to 26.1% (N = 475) in 2013 and 35.7% (N = 751) in 2017 (chi-square P < 0.001, P-for-trend < 0.001). The overall increase between 2008 and 2017 was 97.6%. The above change was solely attributed to the increase in the number of paraclinical investigations during the regular “morning” shift (Figure 5); 145 children performed paraclinical examinations between hours 08:00 and 14:00 in 2008 (representing 38.2% of the total number of paraclinical investigations), 209 in 2013 (44% of paraclinical investigations), and 386 in 2018 (51.4% of paraclinical investigations) (chi-square P < 0.001, P-for-trend < 0.001). From 2008 to 2017, the percentage of paraclinical investigations during the “morning” shift increased by 134%. As a consequence, the respective figures for paraclinical investigations during “afternoon” and “night” shift decreased (P-for-trend < 0.001 for the “afternoon” shift and P-for-trend < 0.001 for the “night” shift) (Figure 5).

The number of children admitted to the hospital remained constant among the three surveys; 313 (18%) of those attending the ED were admitted in 2008, 332 (18.2%) in 2013, and 399 (19%) in 2017 (chi-square *Ρ* = 0.704, P-for-trend = 0.426).

## 4. Discussion

The primary hypothesis of the present study was that the economic crisis would affect the demand for health care services by increasing the number of attendances to the pediatric ED of a large teaching hospital. Similar to other studies [20–22], we have demonstrated an overall increase in the demand for pediatric services by around 21%, before and ten years after the onset of the crisis (2008-2017). This finding was expected, since the economic crisis increases unemployment and reduces incomes, thus leading to serious financial constraints. The association between poor economic status, increased childhood morbidity, and increased demand for pediatric healthcare services has been previously described [28, 29].

We found that the increase in the number of cases attending the ED was solely attributed to children of Greek origin (Figure 2); this is an important finding because it shows that the economic recession affected mainly the indigenous population rather than immigrants or other minor ethnic groups. Indeed, the number of Roma children who visited the ED did not increase throughout the study period, probably because this population (which has already been regarded as socially disadvantaged in Greece) continued to have the same benefits and access to the public healthcare system. On the other hand, the number of children of immigrants attending the ED decreased from 2008 to 2017; this could be explained by the fact that, as the economic crisis arose, a significant proportion of immigrants moved to other countries or their country of origin.

As expected, the increase in the attendances in the ED was more prominent for those living in urban areas (i.e., Regional Unit of Achaia) rather than rural ones (Table 1); this could be explained by the fact that during periods of economic crisis the large cities are usually affected more severely compared to the countryside. In addition, since our hospital is situated in a major city, it could be that those that live far from the hospital could not afford a hospital transfer for minor issues. Of note, the number of patients transferred by ambulance has not been increased which potentially implies that the severity of the reasons leading to a hospital visit has not changed.

Another interesting finding was that the overall increase of ED visits during the economic crisis was mainly driven by the increase in the number of children aged more than five years (Figure 1). Primary pediatric care in Greece is traditionally based on the private sector, meaning that the majority of children have access to a private paediatrician who remains their primary healthcare provider until adolescence. Therefore, the increase of ED visits of older children may reflect the fact that their parents could not afford to consult their private doctor for what they perceived as minor or less severe health issues. In support of the above argument, the increase in the number of ED attendances was due to fever or respiratory symptoms (Table 2), with most of the visits occurring during the regular morning shift (for Greece this is from 8 am to 4 pm) and not out of hours (Figure 4). However, since the number of hospital admissions did not increase during the same period, one may speculate that all these health issues could have well been dealt outside the hospital (i.e., in the private pediatric sector). It is also important to note here that the increase in the ED visits during the economic crisis could not be attributed to low vaccination coverage, as free vaccination remained available for all children in Greece despite the ongoing financial crisis. The adequacy of vaccination is also reflected by the fact that hospital admission rates did not increase during this period.

Perhaps the most striking finding of the present study was the marked increase in the number of children who went through laboratory investigations (blood tests or imaging) during their ED visit, an increase which was disproportionate to the overall increase in ED attendances. Interestingly, more laboratory work occurred during the regular morning shifts, which again indicates that minor health issues that could have been dealt with in the primary care sector were eventually managed in the hospital. Also, as the economic recession evolved, there were more court appeals for medicolegal issues requesting financial compensation which might have potentially led the doctors to order more investigations. In any case, this marked increase in the number of paraclinical investigations increases the economic burden to the public health care system from where all the reimbursement comes from and is suggestive of significant gaps in the primary care organization during the years of economic recession in Greece.

Our results did not coincide with those of previous studies that have correlated the economic crisis with increasing mental health problems [5–7]. However, we acknowledge that the causes of ED visits recorded for our study purposes are not always accurate when it comes to mental health issues. For example, it could be that, in a child presented with headache or chest pain and recorded as such on our database, the doctors detected the presence of psychological issues after careful history and clinical examination. Another reason for which we could not assess the possibility of an increase in mental health issues is that there is not a mental health specialist regularly present in the emergency department of our hospital.

We acknowledge that our study as a pragmatic one has several limitations including its retrospective nature. Moreover, we have assessed only a particular sample of the total attendances and not all of them. However, this sample was large and representative of the total population that attended the pediatric ED of our hospital. Investigating the whole population would perhaps be unrealistic and extremely time-consuming given the fact that during the study period as well as at present there is no electronic medical records system in place. In addition, classification of diagnosis via the ICD-10 system was recorded throughout the study periods due to administrative constraints and therefore not used for data categorization. We also acknowledge that the severity of certain infections, such as influenza, for example, or climate conditions could have changed throughout the consecutive years, although both our local laboratory data and national surveillance data (available at www.keelpno.gr) do not support this assumption. It is therefore understandable that no causality can be directly proven solely from this study, but we can instead comment on the associations observed. Finally, although the study was conducted in only one institution, we believe that the results could be representative for the whole country because this is a tertiary hospital, covering for a large geographical area, and serves approximately one-tenth of the total population in Greece.

## 5. Conclusions

In conclusion, the results of the present study demonstrate an increase in the demand for pediatric hospital services throughout the economic crisis in a large tertiary hospital in Western Greece without a similar increase in the rates of hospital admissions. This might suggest an increase in the number of people in need due to the economic recession as well as organizational issues of the primary care system. Moreover, the increase in the number of visits for minor conditions and the concomitant increase in laboratory procedures placed a financial strain on the paediatric emergency department of our large tertiary hospital. All these issues should be carefully addressed so that both secondary and tertiary care are appropriately delivered and their recourses are well distributed especially in the era of the economic crisis.

## Figures and Tables

**Table 1:** Place of residence of ED attendees.

	**2008**	**2013**	**2017**	**P**	**P-for-trend**
**Achaia** ^a, b^	1277 (73.5)	1429 (78.4)	1757 (83.6)	< 0.001	< 0.001
**Ilia** ^b^	73 (4.2)	76 (4.2)	93 (4.4)	0.909	0.720
**Aetolia-Acarnania** ^b^	109 (6.3)	112 (6.1)	130 (6.2)	0.987	0.920
**Other** ^c^	279 (16.0)	206 (11.3)	121 (5.7)	< 0.001	< 0.001

Data are number of cases with percentages in parentheses. Statistical significance was calculated by chi-square test.

^a^  Local Regional Unit; ^b^  regional units of the region of Western Greece; ^c^  refers to children of whom families were visiting the region of Western Greece at the time of ED presentation.

ED: emergency department.

**Table 2:** Causes of ED visits.

	**2008**	**2013**	**2017**	**P**	**P-for-trend**
**Fever**	461 (26.5)	511 (28.0)	617 (29.4)	< 0.001	< 0.001
**Respiratory symptoms **	244 (14.0)	300 (16.5)	398 (18.9)	< 0.001	< 0.001
**Gastrointestinal symptoms **	107 (6.2)	113 (6.2)	124 (5.9)	0.914	0.732
**Cardiovascular symptoms **	17 (1.0)	15 (0.8)	21 (1.0)	0.828	0.919
**Urogenital symptoms**	20 (1.2)	21 (1.2)	24 (1.1)	0.999	0.976
**Musculoskeletal symptoms**	13 (0.7)	14 (0.8)	12 (0.6)	0.814	0.902
**Skin disorders**	21 (1.2)	23 (1.3)	24 (1.1)	0.798	0.991
**Intoxications, trauma**	20 (1.2)	24 (1.3)	29 (1.4)	0.807	0.524
**Scheduled visits ** ^a^	126 (7.2)	155 (8.5)	233 (11.1)	< 0.001	< 0.001

Data are number of cases with percentages in parentheses. Statistical significance was calculated by chi-square test.

^a^  refers to children visiting the ED to perform scheduled paraclinical investigations or to obtain various health certificates.

ED: emergency department.

## Data Availability

All data used to support the findings of this study are included in the article.
